# Microbes and Their Products for Sustainable Human Life

**DOI:** 10.3390/biom15081168

**Published:** 2025-08-15

**Authors:** Ranjit Gurav, Shashi Kant Bhatia

**Affiliations:** 1 Sustainability Cluster, School of Advanced Engineering, UPES, Dehradun 248007, Uttarakhand, India; rnjtgurav@gmail.com; 2 Department of Biological Engineering, College of Engineering, Konkuk University, Seoul 05029, Republic of Korea; 3 Institute for Ubiquitous Information Technology and Applications, Seoul 05029, Republic of Korea

Microorganisms are the unseen architects of sustainability, intricately woven into the fabric of human survival and planetary balance. While a small fraction are pathogenic, the vast majority of microbes are indispensable allies, forming the cornerstone of food production, environmental restoration, health maintenance, and industrial biotechnology. This Special Issue, “Microbes and Their Products for Sustainable Human Life,” brings together a collection of pioneering studies that underscore the diverse and transformative roles of microbes in our mission towards a healthier and more sustainable world (Figure 1).

Microbes’ role in the valorization of agrowaste into valuable products is gaining research attention. Karirat et al. demonstrated that using *Bacillus tequilensis* PS21, broken riceberry rice and soybean meal agroindustrial byproducts could be utilized to synthesize EPSs with potent antioxidant, antimicrobial, and anticancer properties. Optimization through response surface methodology yielded EPSs at 39.82 g/L, exhibiting a molecular weight of 11,282 Da and amorphous structural properties beneficial for biological applications. This research not only exemplifies waste-to-wealth principles but also showcases how low-cost substrates can support a sustainable EPS production model and, in turn, applications in cosmetics, food, and pharmaceuticals. Apart from individual strains, understanding the ecological dynamics of microbial consortia is crucial, particularly in complex fermentations. Li et al. explored the role of multiple enrichments in Baijiu production, reporting that successive rounds of enrichment significantly altered both metabolite profiles and microbial communities within pit mud ecosystems. Notably, the enrichment led to increased desirable flavor compounds such as caproic and butyric acid, the enhanced levels of which were linked to shifts in dominant genera such as *Clostridium* and *Caproiciproducens*, highlighting how targeted community engineering can optimize traditional fermentation processes. One other study in this issue highlights innovative methods to enhance microbial metabolite production using corn steep liquor hydrolysate. In the case of *Cordyceps militaris*, supplementing the medium with hydrolyzed corn steep liquor significantly improved cordycepin yield. Continuing with fermentation-based innovations, the development of functional beverages represents a growing trend in microbial applications. For instance, the fermentation of licuri kernel extract using water kefir grains yields a probiotic-rich beverage with antioxidant potential. The study by de Carvalho Alves et al. emphasizes the potential of underutilized plant resources in the development of functional foods through microbial processes. These foods are tightly linked to the expanding field of probiotics and gut health. The next set of studies reveals how specific microbial strains and their products offer promising health benefits beyond nutrition. One study on the exopolysaccharide Galacan produced by *Agrobacterium* sp. FN01 demonstrated notable antihyperglycemic and antiaging effects. It enhanced the growth of *Lactobacillus brevis* and modulated telomerase activity, suggesting potential for metabolic disease management in the zebrafish model. In another key study, *Lactobacillus crispatus* CCFM1339 was shown to inhibit vaginal dysbiosis by modulating immune factors and maintaining mucosal balance in mice. These results support the development of next-generation probiotics for women’s health. Additionally, the modulation of gut microbiota by sialic and polysialic acid showed a significant enrichment of *Faecalibacterium* and *Bifidobacterium*, along with elevated SCFA levels. These results further underscore the prebiotic role of microbially derived polysaccharides. Beyond supporting health maintenance, microbial products are increasingly being investigated for their disease-modifying potential, especially in the context of cancer and metabolic disorders. Breast cancer (BC), the most diagnosed cancer in women, is associated with distinct bacterial populations compared to healthy tissue. A study by AlDawsari et al. investigated how metabolites from a triple-negative BC cell line (MDA-MB-231) affect *Pseudomonas aeruginosa*, a breast-resident bacterium linked to oncogenesis. Conditioned media (BC-CM) from BC cells suppressed bacterial growth, caused morphological changes, and significantly altered the bacterial metabolic profile. Notably, BC-CM induced the production of spliceostatin (FR901464), a known antitumor metabolite, along with other compounds that may influence cancer progression. Similarly, addressing lifestyle-related conditions, Zabolotneva et al. examined how pentadecylresorcinol supplementation influenced the gut microbiota in mice on a high-fat diet. The results indicated a shift towards beneficial bacterial taxa, including *Akkermansia muciniphila*, and a reduction in glucose levels, showcasing a microbe-modulating approach to metabolic syndrome. This metabolic modulation is in accordance with additional findings on gut microbiota recovery, highlighting the role of probiotic strains in restoring intestinal health following external disruptions. Two *Enterococcus faecium* isolates were shown to reverse antibiotic-induced gut dysbiosis, restoring beneficial microbial populations and short-chain fatty acid production. These isolates also demonstrated antioxidant and immune-modulating properties, suggesting possible use in functional food applications. It ought to be noted that the broader applications of microbes are not limited to food and health. Synthetic biology and omics technologies are unlocking even more dimensions of microbial functionality. Mantzouridou et al. explored the production of phytoene using engineered microbial systems, revealing the hidden biosynthetic potential of microbial hosts. By integrating multi-omics tools, the study offers a blueprint for the synthetic biology-driven development of microbial pigments with industrial relevance. On a similar front, Qiao et al. employed imaging mass spectrometry and genome mining to identify antimicrobial peptides such as pediocin PA-1 and penocin A produced by *Pediococcus acidilactici* in competitive environments. This work not only highlights microbial responses to environmental stimuli but also suggests novel avenues for natural biopreservatives. Light-driven fermentation optimization also represents a current trend in microalgal biotechnology. Li et al. conducted transcriptomic and metabolomic profiling of *Chlorella pyrenoidosa* under different light conditions and found that red and blue light combinations significantly promoted growth and lipid biosynthesis. These insights pave the way for enhancing biofuel production through targeted light quality adjustments, thereby bridging microbial physiology with renewable energy applications.

Collectively, these studies reiterate the central theme of this Special Issue: microbes are no longer just passive players in natural cycles; they are active agents of change in human health, agriculture, energy, and environmental management. The diversity of microbial products—from biopolymers and probiotics to anticancer compounds and nanomaterials—reflects a vibrant, rapidly evolving frontier. As we continue to harness their potential, we must integrate interdisciplinary approaches that blend molecular biology, process engineering, and systems-level understanding. This Special Issue offers a rich tapestry of insights and innovations, reinforcing that a sustainable human future will be inextricably linked to the microbial world.

## Figures and Tables

**Figure 1:**
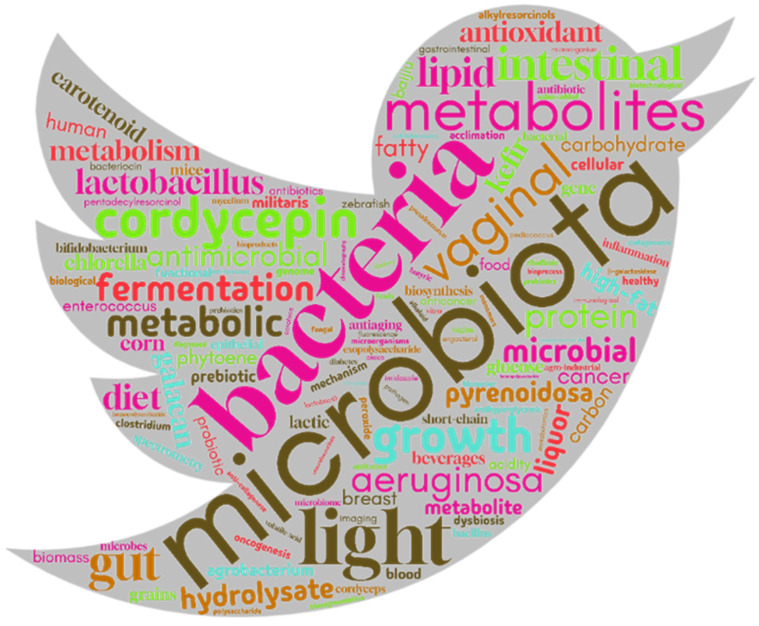
Word cloud of keywords from articles published in this Special Issue. The most frequent words are displayed in a larger font.

